# LFDNN: A Novel Hybrid Recommendation Model Based on DeepFM and LightGBM

**DOI:** 10.3390/e25040638

**Published:** 2023-04-10

**Authors:** Houchou Han, Yanchun Liang, Gábor Bella, Fausto Giunchiglia, Dalin Li

**Affiliations:** 1 School of Computer Science, Zhuhai College of Science and Technology, Zhuhai 519041, China; 2 College of Computer Science and Technology, Jilin University, Changchun 130012, China; 3 Department of Information Engineering and Science, University of Trento, 38100 Trento, Italy

**Keywords:** hybrid recommendation algorithm, deep learning, gradient boosted decision

## Abstract

Hybrid recommendation algorithms perform well in improving the accuracy of recommendation systems. However, in specific applications, they still cannot reach the requirements of the recommendation target due to the gap between the design of the algorithms and data characteristics. In this paper, in order to learn higher-order feature interactions more efficiently and to distinguish the importance of different feature interactions better on the prediction results of recommendation algorithms, we propose a light and FM deep neural network (LFDNN), a hybrid recommendation model including four modules. The LightGBM module applies gradient boosting decision trees for feature processing, which improves LFDNN’s ability to handle dense numerical features; the shallow model introduces the FM model for explicitly modeling the finite-order feature crosses, which strengthens the expressive ability of the model; the deep neural network module uses a fully connected feedforward neural network to allow the model to obtain more high-order feature crosses information and mine more data patterns in the features; finally, the Fusion module allows the shallow model and the deep model to obtain a better fusion effect. The results of comparison, parameter influence and ablation experiments on two real advertisement datasets shows that the LFDNN reaches better performance than the representative recommendation models.

## 1. Introduction

With the rapid development of the Internet, recommendation systems are being used in various business scenarios [1]. Since the development of recommendation system algorithms in the 1990s, they can be summarized into the following three stages [2].

The first stage is the early stage of development (before 2010). Recommendation systems used the method of “artificial features + linear models”, i.e., the expert systems, which are the typical representative of this period [3]. The main characteristics of this stage are as follows. First, the magnitude of the original input characteristics is between one hundred and ten thousand, which is relatively small compared to the subsequent data volume. The second point is that the processed feature magnitude can be anywhere from ten thousand to one hundred thousand to one million levels. Thirdly, although the model is relatively simple, the parameter space is relatively small, and the actual application performance is high, with good prediction results. Fourth, improving the effectiveness of recommendation systems requires relying on business experts to conduct artificial feature engineering, based on their understanding of the business, and mining effective feature combinations through a large amount of manual experience and data analysis.

The second stage is the accelerated development period (2010–2015). The recommendation systems adopted the method of “automatic feature engineering + linear model” [4]. Typical representative methods include: the FM model proposed in 2010 [5]; the Field-Aware Factorization Machines (FFMs) model proposed in 2014 [6]; the GBDT+LR model proposed in 2014 [7]; Personality Computing in 2014 [8]; and XGBoost and others proposed in 2016 [9].The main features of this stage are as follows. Firstly, a supervised automatic cross-over of second-order and higher-order features allows you to remember various effective feature combinations, that is, learn which feature combinations can be used to better distinguish labels. Secondly, the parameter space where features intersect can be controlled by modifying hyperparameters, such as the length of hidden vectors in the FM model, the number and depth of tree models, and so on. The third point is to conduct joint training and learning by combining low order, second order, and high order, with the main purpose of strengthening the memory of each feature or feature combination in the same space to influence the weight of the prediction results. Fourth, the effectiveness of the recommendation system at this stage has been significantly improved [10]. The training stage requires fewer hyperparameters to be adjusted, making it simpler and more efficient.

The third stage is the deep development period (2016 to present). At this stage, features are mapped into multi-dimensional space and then learned through a multi-layer perceptron. Typical representative methods include: the FNN [11] and the Wide&Deep proposed in 2016 [12]; the DIN proposed in 2018 [13]; and DeepFM proposed in 2017 [14]. The main characteristics of this stage are as follows. First, discrete feature processing has begun to use a large amount of Embedding technology, which can more reasonably express features by reducing the dimensions of data from high to low dimensional spaces, allowing for both compression of the feature space and reasonable representation of discrete features. Secondly, in each stage, in order to reduce the magnitude of the parameter space, use as few parameters as possible to mine out the underlying laws of the data under limited sample conditions. Thirdly, mining the relationship between the context and target, such as designing sequence features to mine the rules of correlation between them and the target. Fourth, using a DNN to mine high-order feature information [15]. The fifth point is to combine low-order features, second-order feature combinations, and high-order feature combinations for joint learning. Low-order feature combinations and second-order feature combinations mainly enhance memory ability, while high-order feature combinations mainly enhance generalization ability [16].

In recent years, more and more researchers are focusing on hybrid recommendation systems. Ensemble learning is used in many models which mainly integrate multiple algorithms and combine the advantages of each algorithm for better classification or prediction results. Ensemble learning achieves better results since the system can combine multiple algorithms to substantially reduce the variance [17]. The core idea of a hybrid recommendation system is the same as that of ensemble learning, combining multiple recommendation algorithms to improve the overall performance. The winning team in the 2016 Netflix Prize competition used the GBDT model to combine more than 500 models in order to integrate the strengths of each algorithm, making it one of the most famous cases of using hybrid recommendations to improve model performance in the history of recommendation systems [18,19]. Wide&Deep learning proposed by Cheng H Tet al. is a hybrid model consisting of wide linear models and deep neural networks [12]. The wide part uses the LR model for strong memory capability, and the deep part is in charge of generalization ability. By fusing the two parts, the Wide&Deep model performs excellent in both logistic regression and deep neural networks and is able to process and memorize a large number of historical behavioral features quickly with strong expressive power. However, the wide part requires artificial feature engineering, which causes a long configuration period.

Guo H, Tang R, Ye Y, et al. proposed the DeepFM model, which can be considered as an upgraded version of Wide&Deep [14]. Similar to Wide&Deep, the DeepFM model also consists of shallow models and deep models, with the following two main differences: the wide linear models replace the LR model with the FM model and share original input features. Compared with the LR model used in Wide&Deep, the FM model has the ability to automatically learn feature intersection, avoiding the artificial feature engineering work in the shallow part of the original Wide&Deep model. The original features of the DeepFM model will be used as common inputs for the FM and deep model parts to ensure the accuracy and consistency of the model features. The disadvantage of this model is that the categorical features with large dimensionality will have many problems in FM second-order feature intersections.

Xu J, Hu Z, and Zou J proposed a personalized product recommendation method based on analyzing user behavior using DeepFM [20]. Firstly, the K-means clustering algorithm is used to cluster the original log data from the perspective of similarity to reduce the data dimension. Then, through the DeepFM parameter-sharing strategy, the relationship between low- and high-order feature combinations is learned from the log data, and the click rate prediction model is constructed. Finally, based on the predicted click-through rate, products are recommended to users in a sequence and fed back. The proposed method achieved a better recommendation effect compared with other newer recommendation methods.

Ma M, Wang G, and Fan T proposed the fDeepFM incorporating deep feature extraction [21]. Firstly, the word features are transformed into low-dimensional dense vectors through the Embedding layer. Then, Doc2Vec is combined to mine item features with contexts, and the two are stitched together as the input to the FM model and DNN model. Subsequently, user features are input to the GRU (Gated Cyclic Unit) model according to different cycles to mine user features. Finally, the results of the FM model, DNN model, and GRU model are combined by linear stitching as the overall output of the fDeepFM model. Experiments were carried out on Movielens-20M and Amazon data sets and reached better performance than the DeepFM.

Wang R, Fu B, Fu G, et al. proposed a DCN model that uses a Cross network to replace the wide part of the Wide&Deep model [22]. The Cross network is an efficient way to apply explicit feature crossover. The DCN model is a deep model that can learn both low-dimensional feature crossing and high-dimensional nonlinear features efficiently without manual feature engineering, requiring very low computational resources. However, the Cross network is bit-wise when doing feature intersection and does not consider the concept of the feature field.

T Lian J, Zhou X, Zhang F, et al. proposed the xDeepFM model; the main idea is to add a CIN layer to the Wide&Deep model [23]. The CIN layer is vector-wise, and the elements belonging to a feature field are considered as a whole during feature crossing. The disadvantage is that the complexity of the CIN layer is usually large, which puts pressure on the model to come online.

Ke GL, Qi M, Finley T, et al. proposed the LightGBM model to resolve the time-consuming problem of the conventional GBDT model with two novel techniques: Gradient-based One-Side Sampling (GOSS) and Exclusive Feature Bundling (EFB) [24]. GOSS can obtain a quite accurate estimation of the information gain with a much smaller data size, and EFB bundles mutually exclusive features to reduce the number of features. LightGBM speeds up the training process of the conventional GBDT model by over 20 times while achieving almost the same accuracy. In this paper, based on the better performance of LightGBM, in order to learn higher-order feature interactions more efficiently, to improve the interpretability of the recommendation algorithm model, and to distinguish the importance of different feature interactions better on the prediction results of the recommendation algorithm, we design a hybrid recommendation model LFDNN based on the FM model, LightGBM, and deep neural network. First, LightGBM is used to perform feature selection and feature cross. It converts some of the numerical features into a new sparse categorial feature vector, which is then added inside the feature vector. This part of the feature engineering is learned in an explicit way, using LightGBM to distinguish the importance of different features. The model we proposed consists of shallow networks and deep neural networks in parallel. The two networks work independently. Finally, a Fusion layer is passed through.

In summary, our work makes the following contributions:

(1) We introduce the deep neural networks to recommendation algorithms to learn higher-order feature interactions more efficiently.
(2) The LFDNN proposes a novel method for distinguishing the importance of different feature interactions.

This paper is organized as follows: Section 2 describes the proposed light and FM deep neural network (LFDNN) model. Section 3 provides experimental results. We draw some discussions and conclusions in Section 4.

## 2. Light and FM Deep Neural Networks (LFDNN) Model

**Figure 1:**
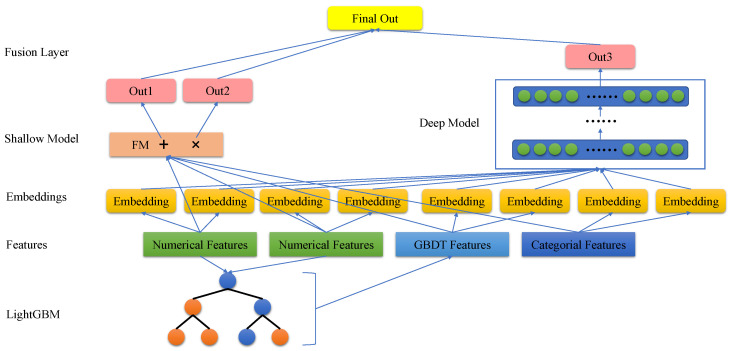
LFDNN model structure diagram.

The network structure diagram of the proposed LFDNN model is shown in Figure 1.

### 2.1. LightGBM Module

**Figure 2:**
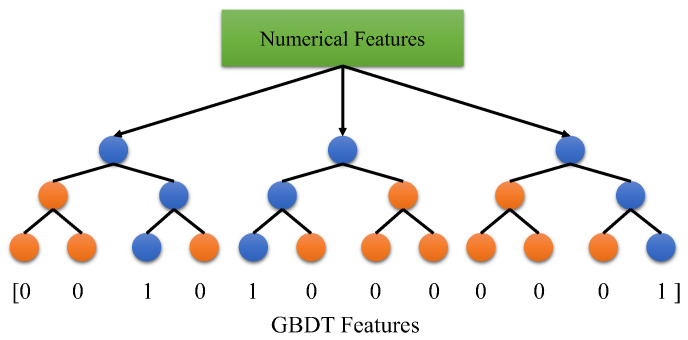
New feature vector-generating process.

Deep neural networks are partially good at handling sparse category features but not dense numerical features, and gradient boosting decision trees are good at handling dense numerical features but not sparse category features [25], so the feature enhancement hybrid principle can be applied to use the gradient boosting decision tree model to handle numerical features and provide new feature inputs for the LFDNN model. After the numerical features are input into the gradient boosting decision tree, the gain is calculated for splitting and finally goes into the underlying leaf nodes to obtain the classification results. The leaf node vectors of all the subtrees are stitched together to form a sparse category feature vector, which is stitched into the feature data of the LFDNN model, such as the GBDT features in Figure 1. The process of generating a new feature vector from the gradient boosting decision tree is shown in Figure 2.

Common algorithms such as neural networks and logistic regression can be trained in small batches, and the size of the training data is not limited by hardware such as the computer’s CPU and RAM [26]. However, gradient boosting decision trees require the data to be traversed multiple times in a single iteration, and if the entire data is fed into the computer system, hardware such as processors and memory can greatly limit the size of the training data if it is not powerful enough. In real business scenarios, the size of the dataset is extremely large. The engineering requirements cannot be met using ordinary gradient boosting decision trees. To solve this problem and make gradient boosting decision trees applicable to industrial practice, Microsoft has proposed the LightGBM framework [24], which is used by the LFDNN model to design the LightGBM module.

### 2.2. Embeddings Layer Design

In various business scenarios, category features and ID-type features are mostly encoded using the one-hot encoding method, which is simple to implement. The coded results are saved in an extremely sparse vector which cannot be taken as the input of a deep neural network directly, as the sparse vector will degrade the performance of the network. The major recommendation algorithms with deep neural networks use dimensionality reduction to avoid this problem. Embedding is one of the most common techniques used. Embedding is a very important feature vector. It is more efficient in transferring information than traditional methods such as matrix decomposition. Therefore, Embedding can be stitched together with the input features of the recommendation system and fed into the deep neural network.

The LFDNN model uses the embedding_lookup() function in the Tensorflow framework to implement Embedding. First, the sparse category features are one-hot processed. Then, they are multiplied via a correlation matrix for Embedding. Finally, a dense matrix is generated. The Embedding operation of multiplying a correlation matrix can be seen as a table look-up operation. The Embedding layer used in this model has two features: despite the different lengths of the inputs, the mapped lengths are the same, both being *k*. There is an empirical formula for the initial determination of the *k*-value of the Embeddings layer, as shown in Equation (1).

(1) $$k=\sqrt[{4}]{x},$$

where *x* in the above equation is the initial number of dimensions, and the *k*-values are adjusted in multiples of 2, e.g., 2, 4, 8, 16. In particular, it is important to note that although the numerical features have been converted into sparse category features by LightGBM, the numerical features are still discretized as ID Features. After Embedding, they participate in the crossing of the FM part of the shallow model together with the Embedding of the other sparse category features.

### 2.3. Design of Shallow and Deep Neural Network Modules

Recommendation models can be broadly classified into two types: shallow models and deep models. The common logistic regression and FM models are both shallow models. Logistic regression models can only capture first-order feature information [23], while FM models can learn second-order feature combinations [5]. With the development of deep learning techniques, recommendation algorithm researchers are applying deep neural networks to improve the accuracy of recommendation systems [27].

The shallow module uses the FM model, which implements feature combination, and the output consists of two parts: an Addition Unit and multiple inner product units. In the FM part of the Network line in Figure 1, the plus sign indicates the Addition Unit part, and the output is Out1 in Figure 1. The Addition Unit reflects the first-order information of the features, and the inner product unit reflects the effect of the second-order feature combination on the prediction result. The output of the shallow model part is shown in Equation (2).

(2) $$y_{FM}=\sum_{i=1}^{m}w_{i}x_{i}+\sum_{i=1}^{n}\sum_{j=i+1}^{n}\langle v_{i},v_{j}\rangle x_{i}x_{j}$$

where $y_{FM}$ is the output of the FM model, $w_{i}$ is the weight parameter, $x_{i}$ is the input, and $\langle v_{i},v_{j}\rangle$ is the inner product unit.

The deep neural network module uses a fully connected feedforward neural network with full connectivity between the individual hidden layers [28]. The output of the Embeddings layer is used as the input to the deep neural network part, and ReLU is used as the activation function between the individual hidden layer nodes. The weight parameter matrix of the first layer of the deep neural network is represented using ***W***_0_ and the bias term is represented using ***b***_0_ to obtain the output of the first layer of the deep neural network, as shown in Equation (3).

(3) $$h_{1}=f\left(W_{0}x_{0}+b_{0}\right),$$

where ***h***_1_ is the output of the first layer, *f*() is ReLU, and *x*_0_ is the output of the Embeddings layer.

With the output of the first layer, following the fully connected model, the output of the second layer of the network is shown in Equation (4).

(4) $$h_{2}=f\left(W_{1}x_{1}+b_{1}\right)$$

The output of the subsequent networks follows this recurrence, with the final output being Out3 in Figure 1.

### 2.4. Fusion Layer Design

The common recommendation methods used in industry have their own advantages and disadvantages, and in order to build on their strengths and avoid their weaknesses, hybrid recommendation systems are often used in practice. One of the most important principles is that the weaknesses of each recommendation algorithm can be avoided by combining multiple recommendation algorithms.

**Figure 3:**
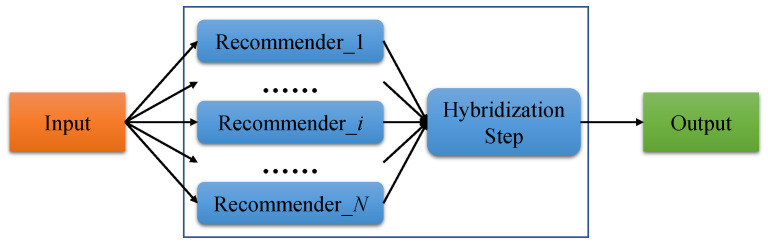
Parallel hybrid recommendation paradigm diagram.

In the parallel hybrid recommendation paradigm, multiple recommendation algorithms exist in parallel in a recommendation system, where the inputs are separated and the results are output independently; finally, these results are fused according to a certain rule-based strategy to return the recommendation results. The specific implementation flow is shown in Figure 3 [29].

There are three specific implementation options. The first is the covariance method. The outputs of multiple recommendation algorithms are placed in a list and returned as one result. The second is a weighting method. This method uses the recommendation results of multiple recommendation algorithms, weighted to obtain a weighted score for each recommendation candidate, and ultimately to rank them. The third method is the branching method. This method develops a recommendation strategy that determines which recommendation algorithm should be used under certain conditions. The development of a recommendation strategy needs to be discussed in the context of the company’s business scenario.

**Figure 4:**
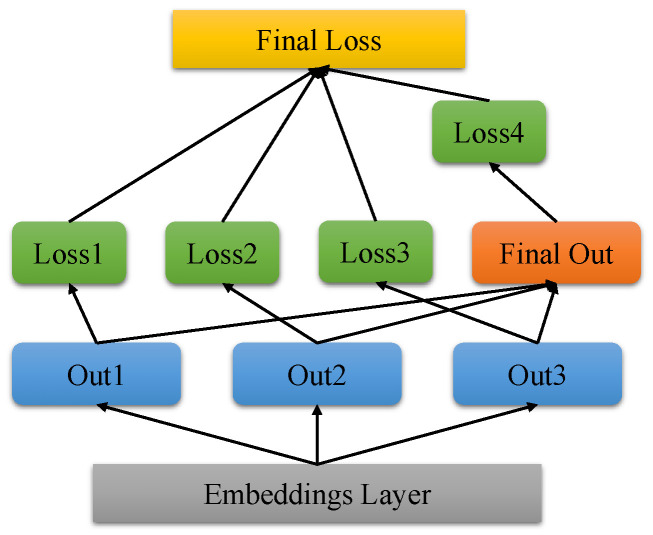
Fusion layer structure diagram.

The Fusion layer in the LFDNN model is designed according to the weighting method in the parallel recommendation paradigm. The role of this part is to allow the shallow module and the deep neural network module to obtain a better fusion effect. The specific design is shown in Figure 4.

**Figure 5:**
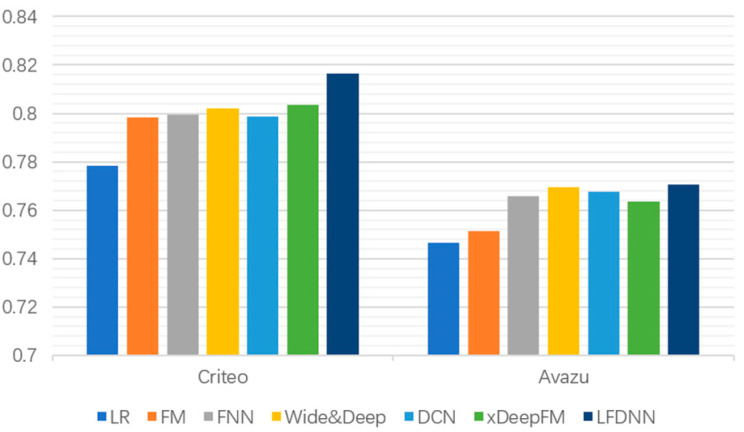
Comparative experimental AUC results graph.

Out1 in Figure 5 is the output of the Addition Unit part of the FM model in the shallow module, Out2 is the output of multiple inner product units of the FM in the shallow model, and Out3 is the output of the deep neural network part. Adding a layer of logistic regression to the above three outputs to change the output into a one-dimensional probability can effectively improve the fusion effect. This is shown in Equation (5).

(5) $$y=sigmoid\left(w1\times Out1+w2\times Out2+w3\times Out3\right)$$

In Figure 5 Loss1 and Loss2 are the losses in the shallow model FM, Loss3 is the partial loss of the deep neural network, and Loss4 is the loss of the final output. The four weight parameters are set as follows: B1 is 0.15, B2 is 0.85, B3 is 0.2, and B4 is 0.2. The calculation of the losses for the whole model of the LFDNN is shown in Equation (6).

(6) Loss=B1×L1+B2×L2+B3×L3+B4×L4

## 3. Experiment Results

In this section we compare the LFNDD with typical commonly used recommendation algorithm models on the datasets, such as Criteo and Avazu, and the better performance of LFDNN is verified.

### 3.1. Experimental Datasets

The recommendation algorithm datasets used in this experiment are: Criteo and Avazu, which are commonly used in evaluating the predictive effectiveness of recommendation models.

Criteo is sourced from Criteo Advertising, and samples are divided into feature information and click information. The feature information is divided into 13 numerical features and 26 categorical features. We divided the 453,798 Criteo samples into a training set and a test set according to a ratio of 5:1.

The Avazu dataset is derived from AD click data from Avazu users’ mobile phones, using information about users’ AD interactions on their mobile devices. The samples are divided into nine numeric and thirteen categorical features. Considering the timing sequence, we treat the earlier samples as the training dataset, and the later samples as the test dataset. In this experiment, 393,288 Avazu samples were divided into training and test sets according to a ratio of 5:1.

To avoid the problem of sample imbalance, the proportion of positive samples in the dataset was allowed to reach about a quarter. Special attention needs to be paid to the fact that some of the samples will have missing data. This situation can cause some difficulty for training. Missing data can be divided into missing categorical features and missing numerical features. For the categorical features, a new category is usually populated, which can be 0, −1, negative infinity, etc. For numerical features, the median is chosen for this experiment to be filled, and this method is insensitive to outliers [22].

### 3.2. Experimental Algorithms and Settings

In order to evaluate the performance of the LFDNN, six classical recommendation models were used for comparison experiments: (1) logistic regression models [23], (2) FM models [1], (3) the neural network FNN model based on the support of factorization machines [29], (4) Wide&Deep [12], (5) DCN, and (6) xDeepFM [30]. (1) and (2) were popular recommendation models before the deep learning era; they can only be trained to learn for the low-order feature combination information in the training data. The LR model can only obtain first-order feature information in application, while the FM model can learn second-order feature combinations. (3) belongs to deep neural network recommendation models from the deep learning era. (4), (5), and (6) are commonly used hybrid recommendation models that combine shallow structures and deep neural networks.

With the LFDNN, the deep neural network module is set to a three-layer network with 300–300–300 neurons per layer, and the dropout rate is set to 0.5, using the Adam optimizer [31]. 

We took Tensorflow as the testing platform with reasonable parameters set to allow the comparison models to achieve the desired performance. All experiments were conducted on a PC equipped with an Intel Core i5-11400 CPU @ 3.20GHz with 16 GB of RAM and an RTX 3070 Laptop GPU.

### 3.3. Comparison Experiments

The performance of the LFDNN model is compared with that of common recommendation algorithm models, and the experimental results are shown in Table 1. In the experiments, we take the AUC (Area Under Curve) [32] and LogLoss [33] as the evaluation criteria.

The AUC evaluates the sorting ability of samples as a whole. The larger the AUC value, the higher the accuracy of model prediction. The LFDNN reaches the highest accuracy on both of the two datasets.

The loss function is a non-negative real value function, which is applied in the training phase for measuring the operation of the algorithm. In this paper, we use the cross-entropy loss function, which measures the difference between the probability distribution and the real distribution of the training results. The closer the two are, the smaller the cross-entropy is. In the experiments, the cross-entropy loss function is first used to evaluate the effect of each sub module in the LFDNN and then the total loss function evaluation value is calculated through the Fusion layer. The LFDNN achieves the best results for both of the two datasets, too.

**Figure 6:**
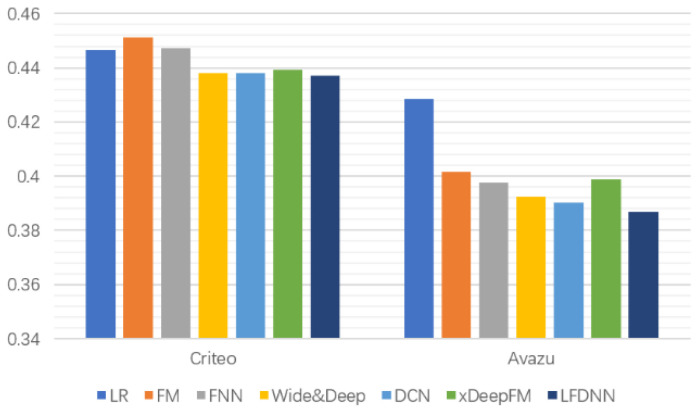
Comparative Experimental LogLoss Results Graph.

In order to observe the analysis more visually, the comparison results are plotted according to the experimental data, as shown in Figure 5 and Figure 6. As shown in Figure 5, the effect of the FM in the shallow model is significantly better than that of LR, indicating that the FM with second-order feature combination information is effective in improving the recommendation effect of the model. Secondly, comparing the shallow model (LR, FM) and the deep model (FNN), the performance of the deep model with the acquisition of higher-order feature information is better than that of the shallow model. Thirdly, the hybrid recommendation model (DCN, xDeepFM) combines the shallow model and the deep model, and then complements some defects of the deep model, thus making the hybrid recommendation model perform better. Finally, it can be found that the LFDNN performs better on two datasets, with 5.34% and 3.23% AUC improvements compared to the experimental results of the worst performing LR model, and a relatively small improvement compared to the three fusion models with the next best performance. Although the performance improvement of the model is only a little, the small improvement can bring great benefits to the service provider in the case of AD recommendation or E-commerce recommendation.

As shown in Figure 6, we can observe the comparison results of each model in terms of LogLoss metric. LogLoss can more intuitively portray the prediction error of the model on the dataset, and the smaller the value, the better the performance of the model. From the figure, we can see that the LFDNN model achieves the best results for both datasets, reflecting that the designed model can indeed bring better performance results. The comparison shows that the performance of the shallow model is not as good as the deep model and the hybrid recommendation model, which is consistent with the results obtained from the AUC metric analysis. This illustrates the importance of higher-order feature interaction information for the recommendation algorithm task.

### 3.4. Parameter Influence Experiments

Normally, in neural networks training experiments, the network shape, number of network layers, and number of neurons in each layer are determined via trial and error for the best parameter settings. Since the deep neural network part of the LFDNN model is responsible for mining the data patterns hidden behind the features, it is necessary to explore a better parameter configuration for it. In these experiments, we explore how to set the parameters of the LFDNN and how the parameters affect the operation of the overall model.

**Figure 7:**
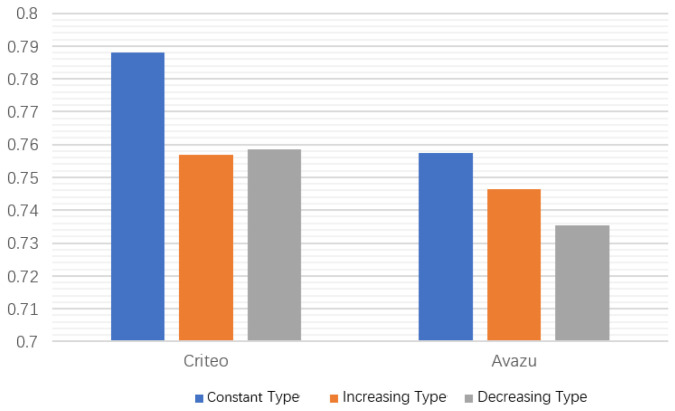
AUC results of network shape experiments.

**Figure 8:**
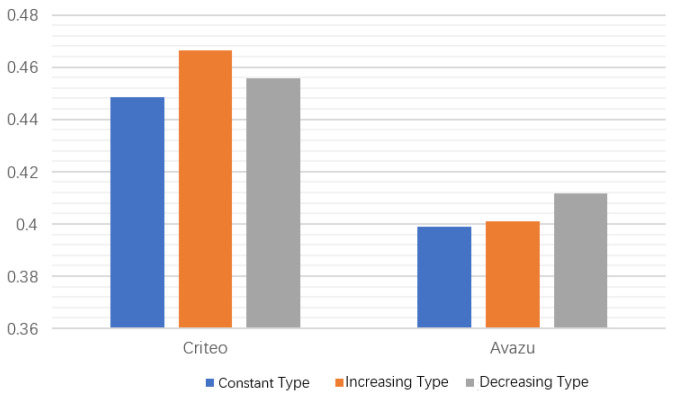
LogLoss results of network shape experiments.

We first test the influence of the network shape on the model by limiting the configuration to three layers and one thousand two hundred neurons. We then test three different network structures: constant, increasing, and decreasing. The constant type of the neural node parameter is set to 400-400-400, the increasing type is set to 200-400-800, the decreasing type is set to 800-400-200, and the dropout rate is uniformly set to 0.5. The experiment results are shown in Figure 7 and Figure 8. As shown in the figures, the constant shape network is superior to the other two shapes in performance in the depth neural network part of the LFDNN.

**Figure 9:**
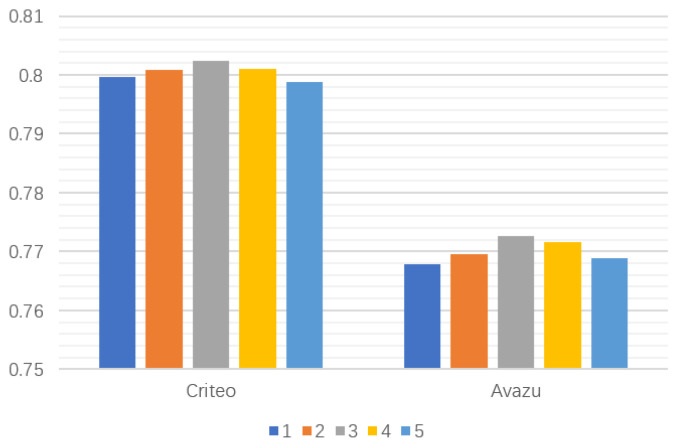
AUC results of network layer experiments (the numbers of 1–5 represent the number of hidden layers in the neural network).

**Figure 10:**
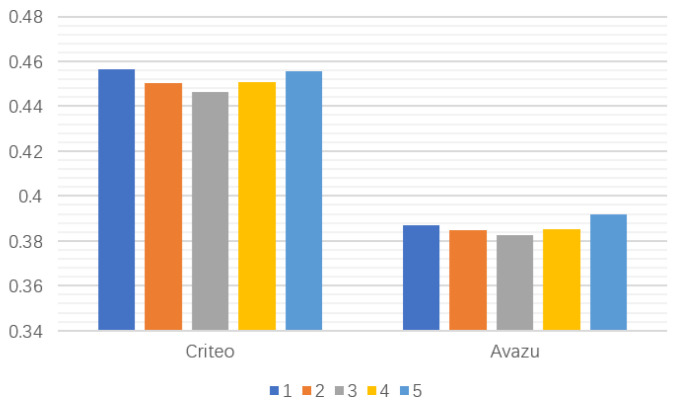
LogLoss results of network layers experiments. (The numbers of 1–5 represent the number of hidden layers in the neural network).

Then, we test the influence of the number of layers of the network on the model. The number of neurons in each hidden layer is set to 400, and then the number of hidden layers in the neural network is set from 1 to 5. The test results of the LFDNN model on the number of network layers are shown in Figure 9 and Figure 10. From the figures, it can be seen that the performance of the LFDNN model improves with the increase in the number of network layers. However, if the number of hidden layers increases, the performance of the model will also decline. This shows that the increase in the number of hidden layers in the deep neural network can enhance the learning ability of the model at an appropriate time, but beyond a certain range, the model will become complex, and the training cost will increase; the model will also become prone to overfitting, and this will limit the ability of the model. The above experiment explored the influence of the number of partial hidden layers of the deep neural network on the performance of the LFDNN model and determined that the optimal hidden layer number is three.

**Figure 11:**
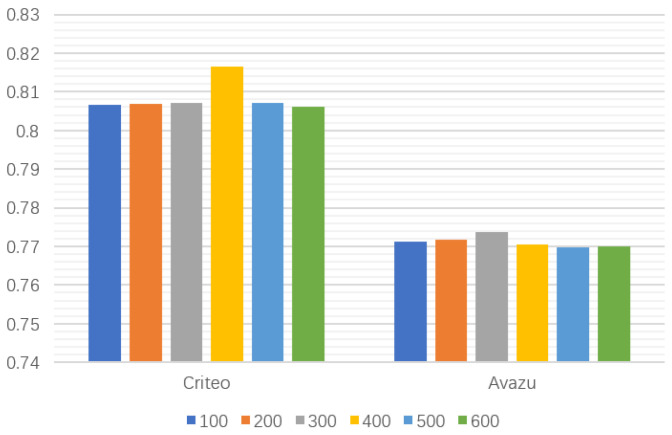
AUC results of network nodes experiments. (The numbers of 100–600 represent the number in each layer of the network).

**Figure 12:**
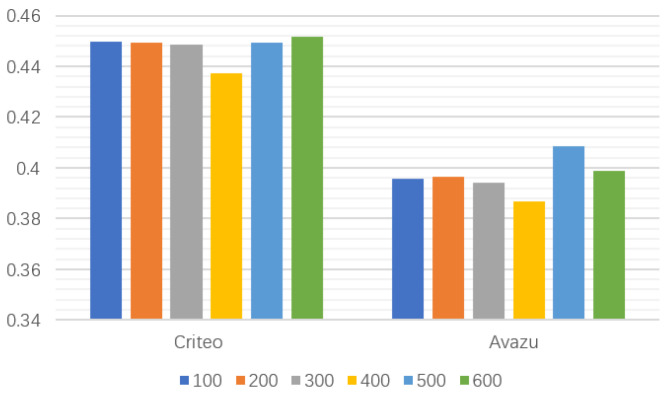
LogLoss results of network nodes experiments. (The numbers of 100–600 represent the number in each layer of the network).

Finally, we explore the appropriate number of nodes in each layer of the network. The number of layers is set as three according to the results of the network layers experiments, and the network shape is constant. The nodes of each layer are set from 100 to 600 in steps of 100, respectively. The experimental results of the LFDNN model on the number of hidden layer nodes are shown in Figure 11 and Figure 12. It can be seen that the AUC and LogLoss obtain better performance at first as the number of nodes increases. However, they begin to decline to a certain extent when the number of nodes exceeds 400. This is because the increase in the number of nodes makes the model more complex, and this make it easy to cause overfitting. Therefore, it is a reasonable choice to set each hidden layer of the LFDNN to between 200–400 neurons.

### 3.5. Ablation Experiments

We further investigated the effects of the Fusion layer and LightGBM module in the LFDNN. The experimental results are shown in Table 2. A is the Fusion layer and B is the LightGBM module. The “√” indicates that the corresponding network is involved.

According to Table 2, first, we can find that the AUC metrics improved by 1.9% and 1.6%, and the LogLoss decreased by 4% and 2.3% while the Fusion layer was involved. This is because a pipelined hybrid recommendation paradigm is applied, where the shallow part and the deep neural network part of the LFDNN are viewed as a whole, and then the output of this part is used as the input of the logistic regression in the Fusion layer; finally, the recommendation results are obtained. It is observed that the outputs of the shallow and deep models can be better fused. Secondly, it can be seen that after using the LightGBM module, the AUC metrics improve by 2.6% and 2.2%, and the LogLoss decreases by 1.8% and 3.9%. This is due to the fact that the module extracts more information from the dense numerical features and adds them to the subsequent inputs. The design improves the ability of the hybrid model to utilize dense numerical features.

## 4. Discussion and Conclusions

Our work proposes the LFDNN, an improved hybrid recommendation model. Firstly, the model uses LightGBM for feature engineering, which can more effectively collect feature combination information, thereby improving the interpretability of the recommendation algorithm model. Then, the model is divided into a shallow network part and a deep neural network part. The shallow network uses an FM model, and the deep part uses a fully connected feedforward neural network. Finally, a Fusion layer is designed to allow the two parts to learn jointly, thereby modeling a hybrid recommendation algorithm model with better overall performance. Compared with classic recommendation models on the two real advertising datasets, the LFDNN achieves better performance, which improves its effectiveness.

The LFDNN also has certain inadequacies. Limited by the features of the composed algorithms, in business scenarios where category features [34] account for the majority, the LFDNN may not be as good as other existing models, and we are continuously working on new technologies, i.e., reinforcement learning, which can be applied to the field of recommendation algorithms if the recommendation system is treated as an agent and the training and updating process of the recommendation system is treated as a cycle of the agent. We will conduct further research on category feature data based on reinforcement learning.

## Figures and Tables

**Table 1:** Comparison experimental results.

Model	Criteo		Avazu	
	AUC	LogLoss	AUC	LogLoss
LR	0.7785	0.4667	0.7464	0.4286
FM	0.7982	0.4513	0.7513	0.4015
FNN	0.7996	0.4473	0.7659	0.3977
Wide&Deep	0.8019	0.4379	0.7695	0.3925
DCN	0.7988	0.4381	0.7678	0.3902
xDeepFM	0.8034	0.4393	0.7636	0.3987
LFDNN	0.8166	0.4372	0.7705	0.3868

**Table 2:** Ablation experimental results.

A	B	Criteo		Avazu	
		AUC	LogLoss	AUC	LogLoss
√		0.7954	0.4452	0.7534	0.4025
	√	0.8010	0.4557	0.7586	0.3961
√	√	0.8166	0.4372	0.7705	0.3868

## Data Availability

The data presented in this study are available on request from the corresponding author. The data are not publicly available due to further research plan.
